# A Physics-Informed Framework for Force-to-Stress Conversion in Small Punch Creep Tests of Creep-Resistant Alloys at High Temperatures

**DOI:** 10.3390/ma19214620

**Published:** 2026-10-30

**Authors:** Manlin Huang, Limin Wei, Yi Cheng, Kaishu Guan

**Affiliations:** 1 School of Mechanical and Power Engineering, East China University of Science and Technology, Shanghai 200237, China; 2 State Key Laboratory of Low-Carbon Thermal Power Generation Technology and Equipments, Harbin 150046, China; 3 Harbin Boiler Company Limited, Harbin 150046, China

**Keywords:** small punch creep test, transfer learning, force-to-creep stress ratio, data interpretation

## Abstract

The small punch creep test (SPCT) requires only a small amount of material, but converting the applied force into an equivalent uniaxial creep stress remains difficult. This study presents a physics-informed framework that uses transfer learning for this conversion in creep-resistant alloys at high temperatures. A Teacher Model is trained on uniaxial creep rupture data to relate rupture life to stress, temperature, and chemical composition. A Mapper Network then estimates the equivalent uniaxial stress from the SPCT force, temperature, and composition, so that the Teacher Model reproduces the measured SPCT rupture life. The physical constraints are this rupture life equivalence and an empirical force-to-stress anchor; the network does not solve a creep constitutive equation. The conversion was examined for P91 steel, 2.25Cr1MoV steel, 1CrMoV steel, and Incoloy 800H, using specimens of 0.5 mm thickness. For these alloys, the converted stresses are compared with uniaxial Larson–Miller curves. The resulting force-to-stress ratio varies with temperature, force, and material within the tested range.

## 1. Introduction

**Figure 1:**
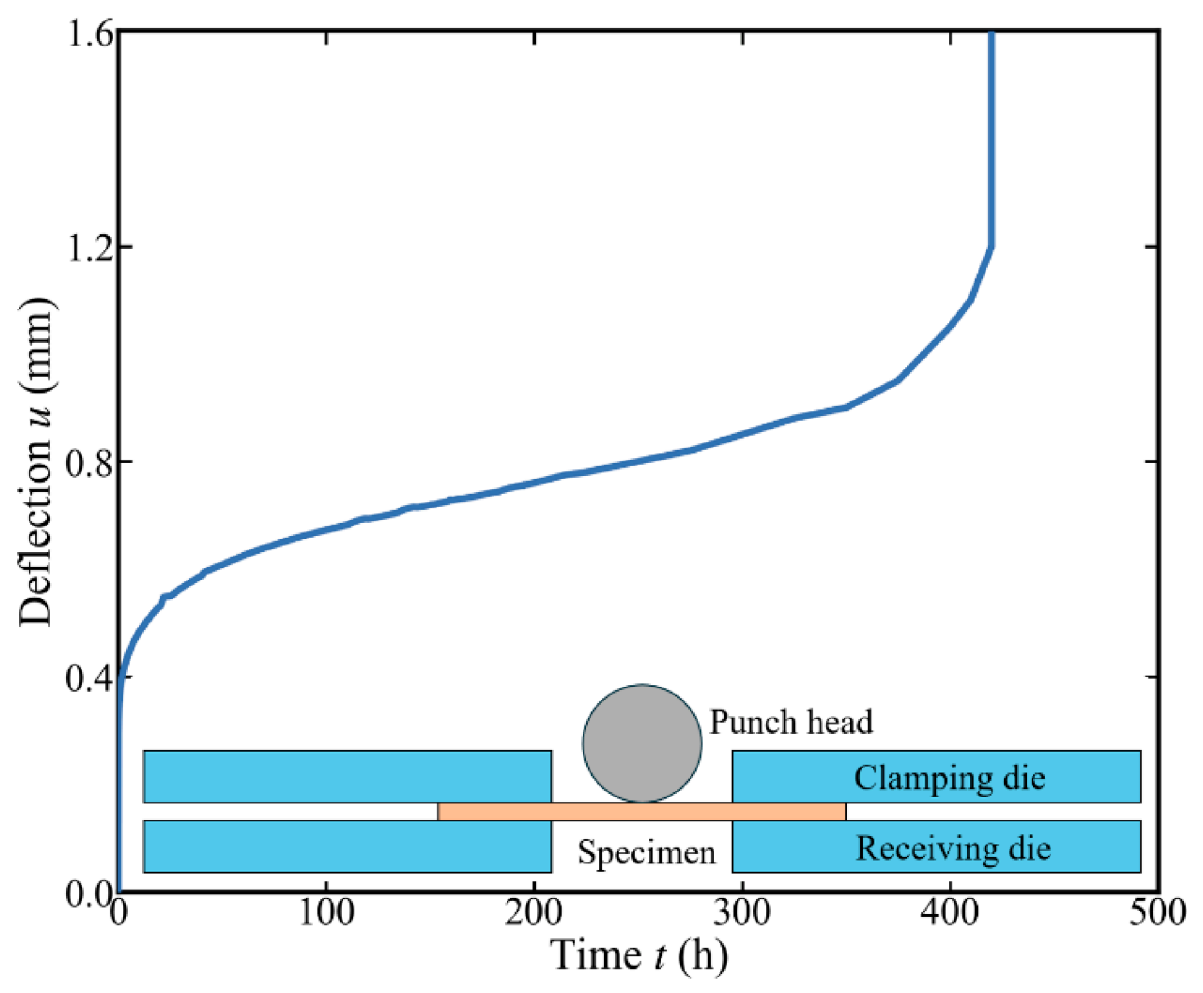
Schematic diagram of small punch creep test setup and deflection–time curve.

The Materials Genome Initiative has elevated high-throughput experimentation to a key role in accelerating the development of advanced materials [1]. Efficient and reliable mechanical characterization is of significant importance in high-throughput experimentation. Small sample testing techniques, such as the small punch test (SPT) [2], indentation test [3], and small tensile test [4], have emerged as highly attractive high-throughput characterization techniques owing to the small amounts of material required for sampling. Among these tests, SPT is an attractive testing technique for the evaluation of creep properties of materials. The SPT specifically used to evaluate the creep properties of materials is commonly named the small punch creep test (SPCT). The SPCT evaluates the creep performance of materials by applying a constant force to a thin disk specimen and recording the evolution of its central deflection over time. Figure 1 illustrates the SPCT configuration together with a typical deflection–time curve. The SPCT specimen has a diameter of no more than 10 mm and a thickness of no more than 0.5 mm. To promote the use of the SPCT, the European Committee for Standardization (CEN) issued the CWA-15627 agreement in 2007 [5], which was later developed into the standard EN-10371 in 2017 [6]. The Society of Materials Science of Japan published an SPCT standard for assessing the remaining life of high-temperature components [7], and a Chinese standard issued in 2025 also detailed SPCT procedures. Converting the SPCT force into equivalent uniaxial creep stress is the core of data interpretation in these standards.

The SPCT force-to-stress conversion methods can be categorized as empirical correlation [6], theoretical analysis [8,9,10,11,12], and inverse finite element analysis [13]. Empirical correlation methods establish simple linear correlations between the SPCT force and a creep stress via statistical regression of extensive experimental data [6,7,14,15]. For instance, the CWA method proposed by CEN introduced a force-to-creep stress ratio with a material-dependent correction factor *k*_sp_ [6], while Holmström et al. [15] further developed deflection-corrected empirical correlations, namely the EFS method and the modified Chakrabarty (MCH) method. However, Tian et al. [16] reported that empirical correlations consider neither the microscopic mechanism nor the nonlinear effect of temperature on creep deformation, resulting in a force-to-stress ratio that shows strong material dependence. Theoretical analysis, relying on the membrane stretching solution of Chakrabarty theory [9], has progressively refined the description of the deformed specimen. Hyde et al. [10] proposed a conical deformation model accounting for thickness variation, and Yang et al. [11] and Zhao et al. [12] further developed spherical–conical geometric models. However, these analytical methods geometrically simplify the deformation of the SPCT specimen and overlook both the non-uniform stress distribution and the initial plastic deformation characteristic of SPCT [17], which prevents them from accurately capturing the localized heterogeneous deformation and microstructural damage evolution. In addition to analytical approaches, finite element method (FEM) simulations were widely utilized to resolve the local stress–strain fields of SPCT specimens. For example, Xie et al. [13] applied FEM to extract Norton creep properties of P91 steel and P92NT steel from SPCT. Although physically meaningful, FEM-based analysis requires the calibration of creep constitutive models and heavily depends on massive iterative computation, leading to excessively long computation times and high computational costs. There is an urgent need for a method that considers the SPCT creep mechanism and is simple to use.

Recently, data-driven machine learning (ML) has offered novel solutions for predicting the mechanical properties of materials. For instance, Xiong et al. [18] predicted the fatigue strength, tensile strength and hardness of steels from chemical compositions and processing parameters. Haghighat et al. [19] employed physics-informed neural networks for inverse identification of constitutive parameters and surrogate modeling in solid mechanics. Gao et al. [20] incorporated chemical compositions and processing parameters into an ML model, and this model successfully predicted the creep rupture life of a Ni-based single-crystal superalloy with high precision. Zhong et al. [21] extracted tensile properties from a force-deflection curve of SPT by using a data-driven method. The above studies not only validated SPT reliability in characterizing mechanical properties but also established a methodological foundation for evaluating more complex high-temperature creep behaviors. ML usually requires large datasets, whereas SPCT data suffer from “small data” limitations.

To address the limited amount of SPCT data, this paper uses uniaxial creep records to train a rupture life model and then transfers that model to SPCT force-to-stress conversion. The central contribution is a physics-informed framework for estimating equivalent uniaxial stress for creep-resistant alloys at high temperatures. In this framework, the equivalent stress is the uniaxial stress that reproduces the measured SPCT rupture life at the same temperature and chemical composition. The effects of temperature, force, and material on the conversion ratio are then examined for the alloys and conditions contained in the dataset. This study does not establish a constitutive creep model, and the reported conversion is not claimed for specimen thicknesses, alloy classes, or temperatures outside those evaluated below.

## 2. Methods

### 2.1. Transfer Learning Framework

**Figure 2:**
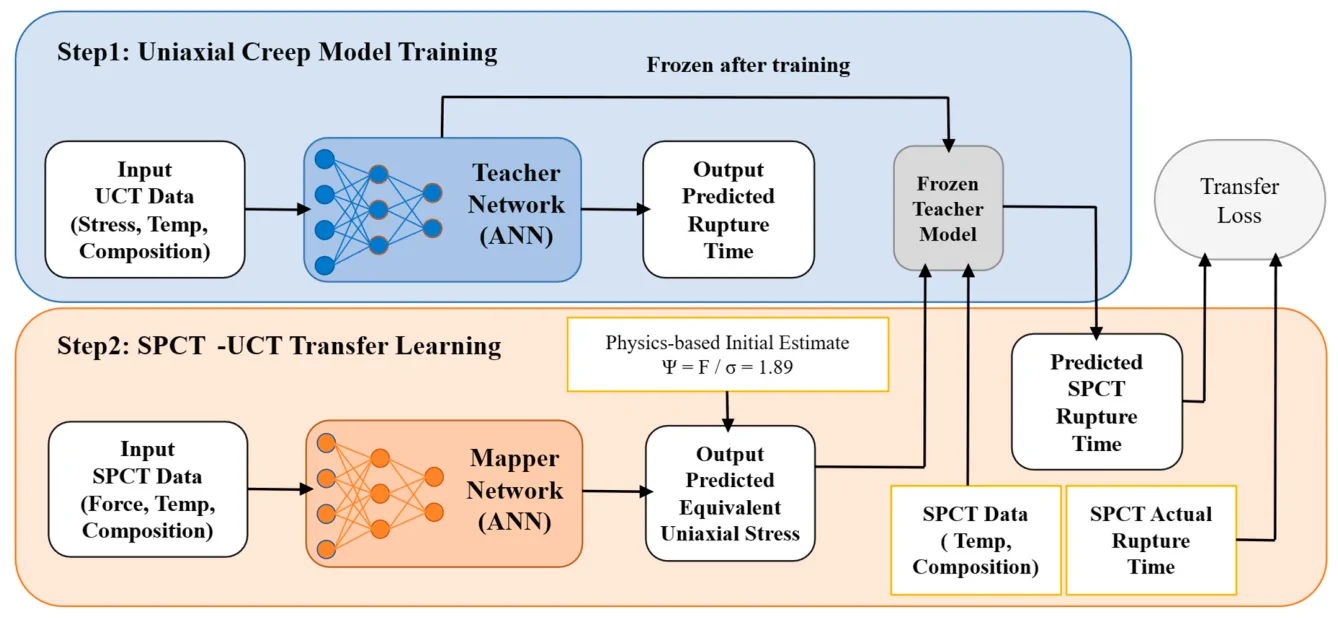
Schematic diagram of the transfer learning framework.

The core concept of this framework was utilizing the abundant uniaxial creep test (UCT) data to train a Teacher Model for capturing the complex nonlinear physical dependence of creep rupture life on the testing temperature, applied stress, and key chemical composition of the material. Subsequently, this model guided the parameter optimization for a Mapper Network. The Mapper Network can predict the equivalent uniaxial creep stress from an SPCT force, testing temperature, and chemical composition. The transfer learning framework includes two steps, as shown in Figure 2. In the first step, we constructed a fully connected deep neural network as a Teacher Model, aimed at learning the nonlinear mapping relationship between the chemical composition of the material, testing temperature, uniaxial stress, and creep rupture life. The second step established a Mapper Network, $g_{\theta_{M}}$, which converted an SPCT force, $F_{sp}$, into an equivalent uniaxial creep stress, $\sigma_{eq}$. The mapping relationship was defined as $\sigma_{eq}\;=\;g_{\theta_{M}}\left(F_{sp},T,C\right)$. Standard EN-10371 suggests that $\sigma_{eq}$ derived from the SPCT is equal to the stress of UCT if the creep rupture time is identical for two kinds of tests at the same testing temperature [6]. The equivalent uniaxial stress is the stress supplied to the Teacher Model with the same temperature and chemical composition, so that the predicted rupture life matches the measured SPCT rupture life. This is the rupture life equivalence in EN 10371 [6].

The Teacher target is $\log_{10}t_{r}$, where $t_{r}$ is the rupture time in hours. Input variables and this logarithmic life are standardized with the mean and standard deviation of the Teacher training split. The Teacher loss is the mean squared error between the network output and the standardized logarithmic life:

(1) $$L_{T}\;=\;\left(1/N\right)\Sigma {\left(\hat{y}\;-\;y\right)}^{2}$$

where y is the standardized value of $\log_{10}t_{r}$.

For the Mapper, the force is entered as F/1000, with F in newtons, while temperature and composition are standardized with the Teacher scaler. The Mapper output $\hat{\sigma }$ remains in megapascals. Its training loss is:

(2) $$L_{M}\;=\;\left(1/N\right)\Sigma {\left[\log_{10}\left({\hat{t}}_{r}\right)\;-\;\log_{10}\left(t_{r,SPCT}\right)\right]}^{2}$$

The conversion of one record proceeds in five steps. (1) Read F, T, the nine concentrations, and the SPCT rupture time. (2) Standardize T and composition with the Teacher training scaler, and replace F with F/1000. (3) Evaluate the Mapper to obtain $\hat{\sigma }$. (4) Pass T, $\hat{\sigma }$, and composition through the fixed Teacher to obtain the predicted rupture life. (5) Repeat with the 20 trained seeds and report the mean and standard deviation of $\hat{\sigma }$. One P91 record illustrates the conversion. The test conditions are 600 °C and 240 N, and the measured rupture time is 420 h. The composition, in weight percent, is Ni 0.30, Al 0.012, Cr 8.93, Mo 0.94, Ti 0.003, Nb 0.067, Si 0.32, C 0.106, and B 0.0004. The 20 Mapper runs give 136.1 ± 4.9 MPa. For the same force, F/1.89 = 127.0 MPa.

To prevent the Mapper Network from falling into physically inexplicable local minima during the initial training process, this study introduced a domain knowledge-based “Warm-start Initialization Strategy” [22]. Appendix G in standard EN-10371 [6] suggests that the force-to-stress ratio is 1.89. Thus, the initial weights of the Mapper Network were configured to yield an output stress with a corresponding force-to-stress ratio close to 1.89. The network architectures and key training hyperparameters used for the Teacher Model and Mapper Network are summarized in Table 1. The Softplus activation function was employed in the output layer of the Mapper Network to ensure that the predicted equivalent uniaxial creep stress remained positive.

### 2.2. Data for Transfer Learning

Approximately 2500 UCT records were obtained from the MatDB online database of the European Commission’s Joint Research Centre [23], including both ferritic/martensitic heat-resistant steels and austenitic stainless steels. This raw database was subjected to rigorous data cleaning and physical constraint filtering [24]. Records were removed when the rupture time, stress, or temperature was missing; when the rupture time was shorter than 10 h; or when the stress was not positive. Duplicate uniaxial records with the same material, temperature, and stress were removed. Finally, 825 records were retained as the source domain dataset. Table 2 summarizes the statistical distribution of the source domain dataset, where SD denotes the standard deviation reflecting the dispersion of each feature. Testing temperature, applied stress, and 9 key chemical elements are input features, and the target is the rupture time. To endow the Teacher Model with reliable physical generalization capabilities, this dataset incorporates a wide span of testing parameters, such as temperatures ranging from 450 °C to 1150 °C, stresses ranging from 4.7 MPa to 900 MPa, and rupture lives spanning from 10 h to 132,550 h. The chemical composition includes the mass fractions of nine alloying elements, such as Cr, Ni, and Al.

The target domain dataset was experimental SPCT data designed to train and evaluate the Mapper Network. This dataset, obtained from specimens with a thickness of 0.5 mm and diameters of 8 mm or 10 mm, comprises both experimental data from our laboratory and from European Commission’s MatDB database, totaling 200 raw samples. For the EN 10371 geometry used with these specimens, the punch diameter is 2.5 mm and the receiving-hole diameter is 4 mm [6]. Clamping force is not an input of the Mapper. Where heat treatment is given in the source records, it is commonly normalizing and tempering or a solution treatment, and it is not used as a network input. To ensure both the reliability of the transfer learning process and the convergence of the physics-informed framework, a rigorous data cleaning and screening strategy was applied to the raw dataset. Following this screening process, 153 SPCT samples were retained as the target domain dataset for subsequent training, validation, and testing of the Mapper Network. Table 3 summaries the SPCT dataset.

Table 2 and Table 3 show that the input and target features span several orders of magnitude. Feeding them directly into the neural network can result in flattened loss function contours, which degrade the convergence efficiency of the gradient descent algorithm. To eliminate the errors induced by this large span of input and target features, the Z-score standardization method was employed to linearly transform all input feature vectors and target variables. The Z-score standardization is expressed as [25]:

(3) $$x^{*}\;=\;\frac{x\;-\;\mu_{x}}{\sigma_{x}}$$

where $\mu_{x}$ is the mean of the feature, and $\sigma_{x}$ is the standard deviation of the feature. Thus, all input and target features were standardized to have a mean of zero and a standard deviation of one. The predictions of models were inversely transformed back to the physical scale during evaluation [26].

### 2.3. Validation of Transfer Learning

The 825 samples in the source domain were randomly partitioned into training, validation, and testing sets at an 8:1:1 ratio. To estimate model reliability and robustness, the training and validation processes were repeated 20 times for the Teacher Model, each with different random seeds. For the Mapper Model, the dataset partitioning and training strategies were consistent with those employed for the Teacher Model. The training and validation losses at every epoch were discussed first, and then the accuracy of the transfer learning farmwork in SPCT force-to-creep stress conversion was verified by using the creep data of P91 steel, 2.25Cr1MoV steel, 1CrMoV steel, and Incoloy 800H alloy. These 4 materials were from the target domain. Thus, the predicted creep stresses were compared against the Larson–Miller master curve, which was established from UCT data in the source domain. The Larson–Miller parameter (LMP) is expressed as:

(4) $$LMP\;=\;T(C\;+\;\log_{10}t_{r})\;$$

where T is the absolute temperature (*K*), $t_{r}$ represents the creep rupture time (*h*), and the material constant *C* takes the empirical value of 20 [27].

### 2.4. Application of Transfer Learning

Based on the validated transfer learning framework, the key factors governing the conversion of SPCT force to creep stress were systematically investigated. A global interpretability analysis of the Mapper Network was conducted using the SHAP method. The Shapley values of the input features, specifically SPCT force, temperature, and chemical composition, were calculated to quantify their influence on the predicted creep stress. Then, the fundamental mechanisms for the dependence of the force-to-creep stress ratio on force, temperature, and material were further explored.

## 3. Results and Discussion

### 3.1. Accuracy of Teacher Model

**Figure 3:**
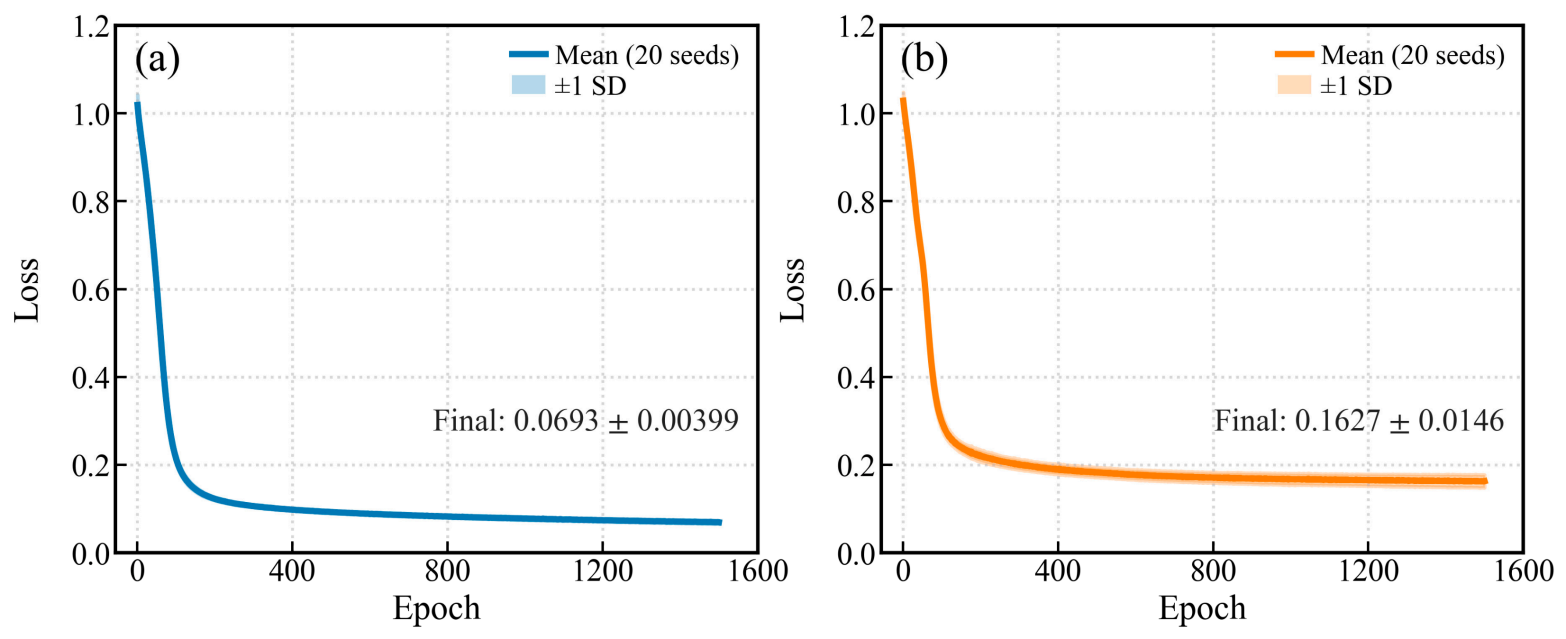
Loss convergence curve of Teacher Model: (**a**) training and (**b**) validation.

Figure 3 shows the mean training and validation losses across 20 runs of the Teacher Model, with the shaded region representing the ±1 standard deviation. It can be observed that the mean loss value decreases rapidly in the initial stage and converges after approximately 1500 epochs, with the final loss stabilizing at around 0.07 and 0.16, respectively, for training and validation. The remarkably narrow shaded regions for both training and validation loss curves indicate consistent convergence behavior across different random initializations, demonstrating the robustness of the Teacher Model against randomness.

**Figure 4:**
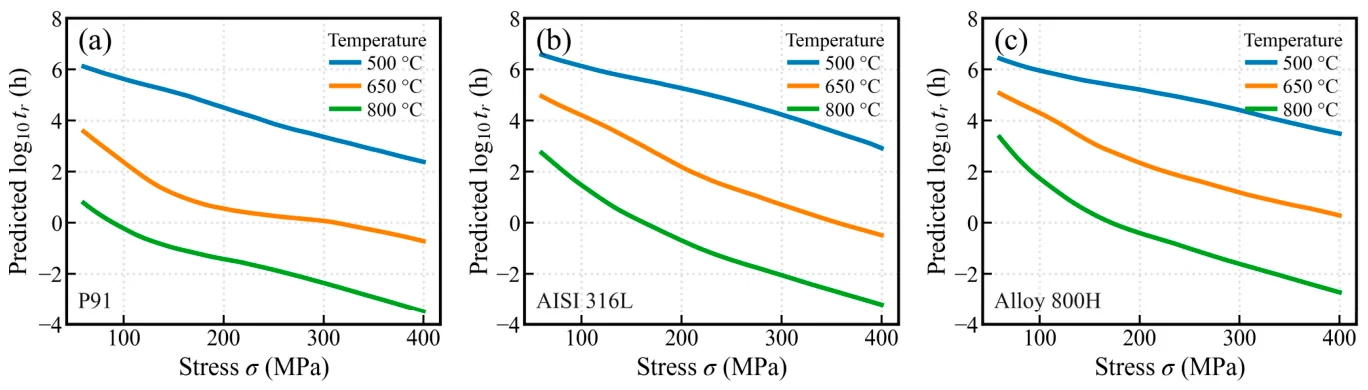
Stress–creep rupture life relation predicted by Teacher Model for three materials: (**a**) P91 steel, (**b**) stainless steel 316 L, (**c**) Incoloy 800 H alloy.

Figure 4 illustrates the relationship between creep stress and creep rupture life as predicted by the Teacher Model. It is evident that, for all three materials, the predicted creep rupture life decreases monotonically with both increasing applied stress (at a given temperature) and increasing temperature (at a given stress). Importantly, no physically anomalous behavior (e.g., longer life at higher stress or higher temperature) is observed, confirming the predictions.

**Figure 5:**
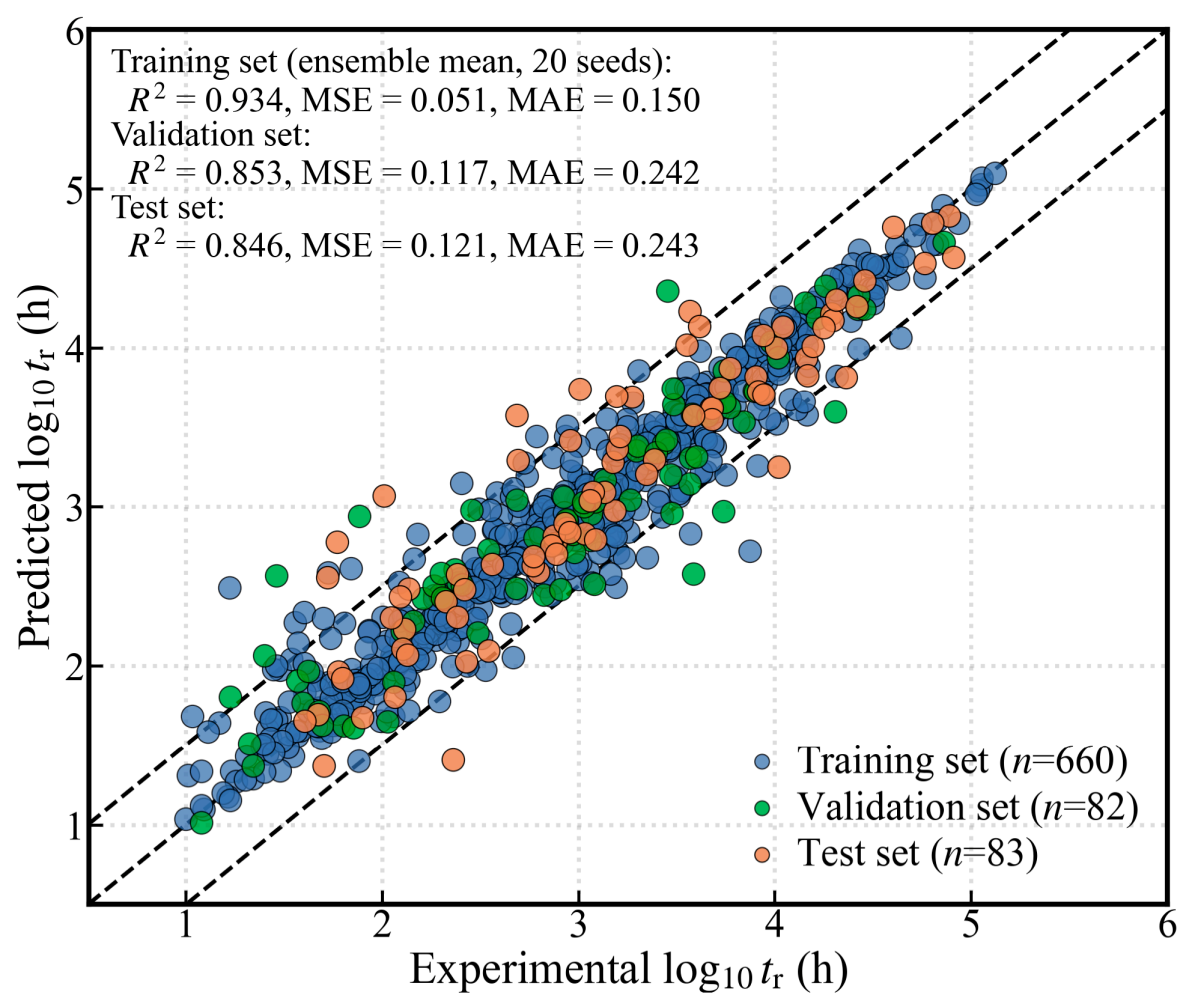
Performance of Teacher Model in predicting UCT creep rupture life. The two parallel dashed lines indicate the 95% error bounds, given by ±1.96 standard deviations of the residuals pooled over the training, validation, and test sets.

The Teacher Model is physically reasonable. The curves are calculated at 500 °C, 650 °C, and 800 °C to represent a low, intermediate, and high temperature in the uniaxial range learned by the Teacher. The SPCT records converted in this study lie between 550 °C and 725 °C. Figure 5 illustrates the predictive performance of the Teacher Model on the source domain dataset, where the horizontal axis represents the experimental creep rupture life, and the vertical axis represents the predicted average rupture life of 20 runs. Ideal predictions should lie near the 45° diagonal line.It can be seen from Figure 5 that most data points are distributed close to the diagonal line, indicating that the Teacher Model possesses strong predicting capability. The coefficient of determination (*R*^2^) of the training sets is 0.934, the MSE is 0.051, and the MAE is 0.160. For testing sets, the *R*^2^ is 0.846, MSE is 0.121 and MAE is 0.243. Furthermore, the 95% error bounds shown in Figure 5 indicate that most predictions fall within a reasonable error range. This result suggests that the Teacher Model has effectively learned the key physical correlation features underlying the high-temperature creep behavior of materials. Thus, it can serve as a reliable physics surrogate, which provides a stable physical guidance foundation for the subsequent SPCT Mapper Network.

### 3.2. Accuracy of Mapper Network

**Figure 6:**
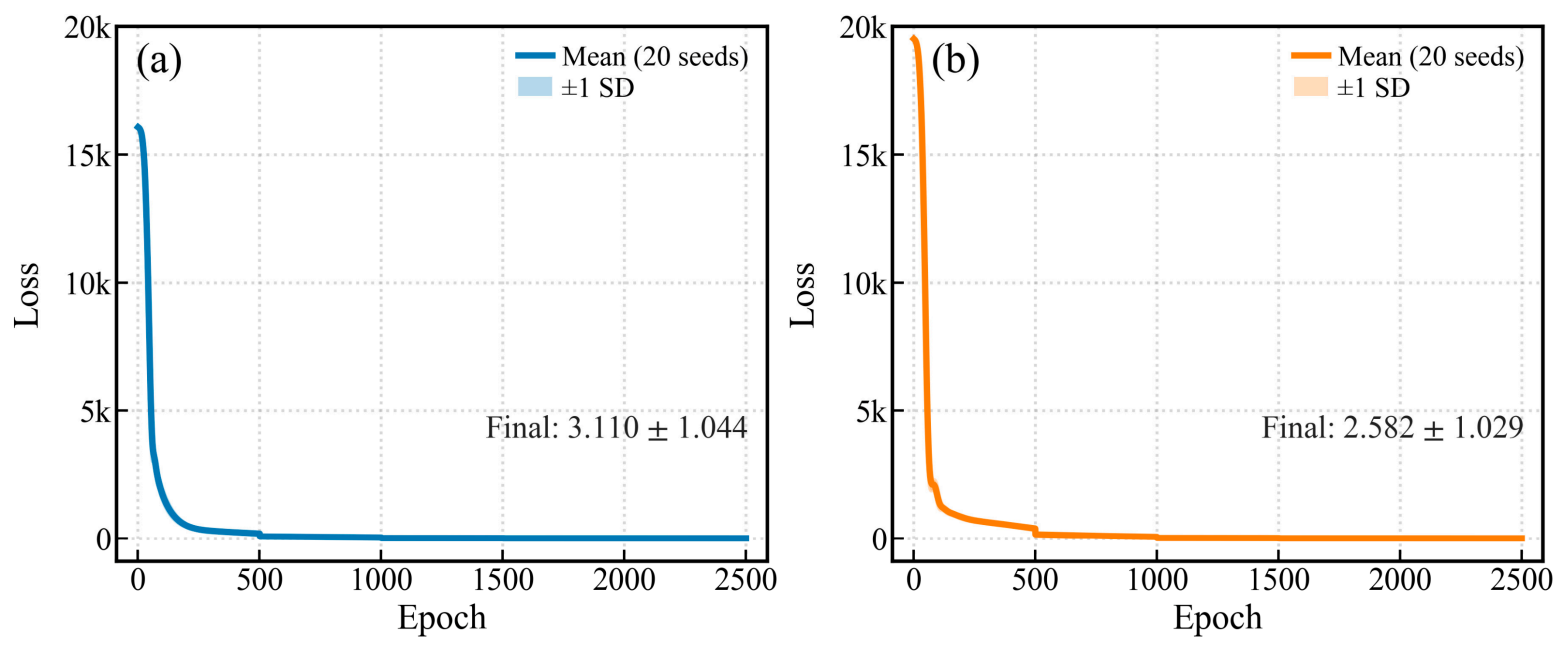
Loss convergence curve of Mapper Network: (**a**) training and (**b**) validation.

**Figure 7:**
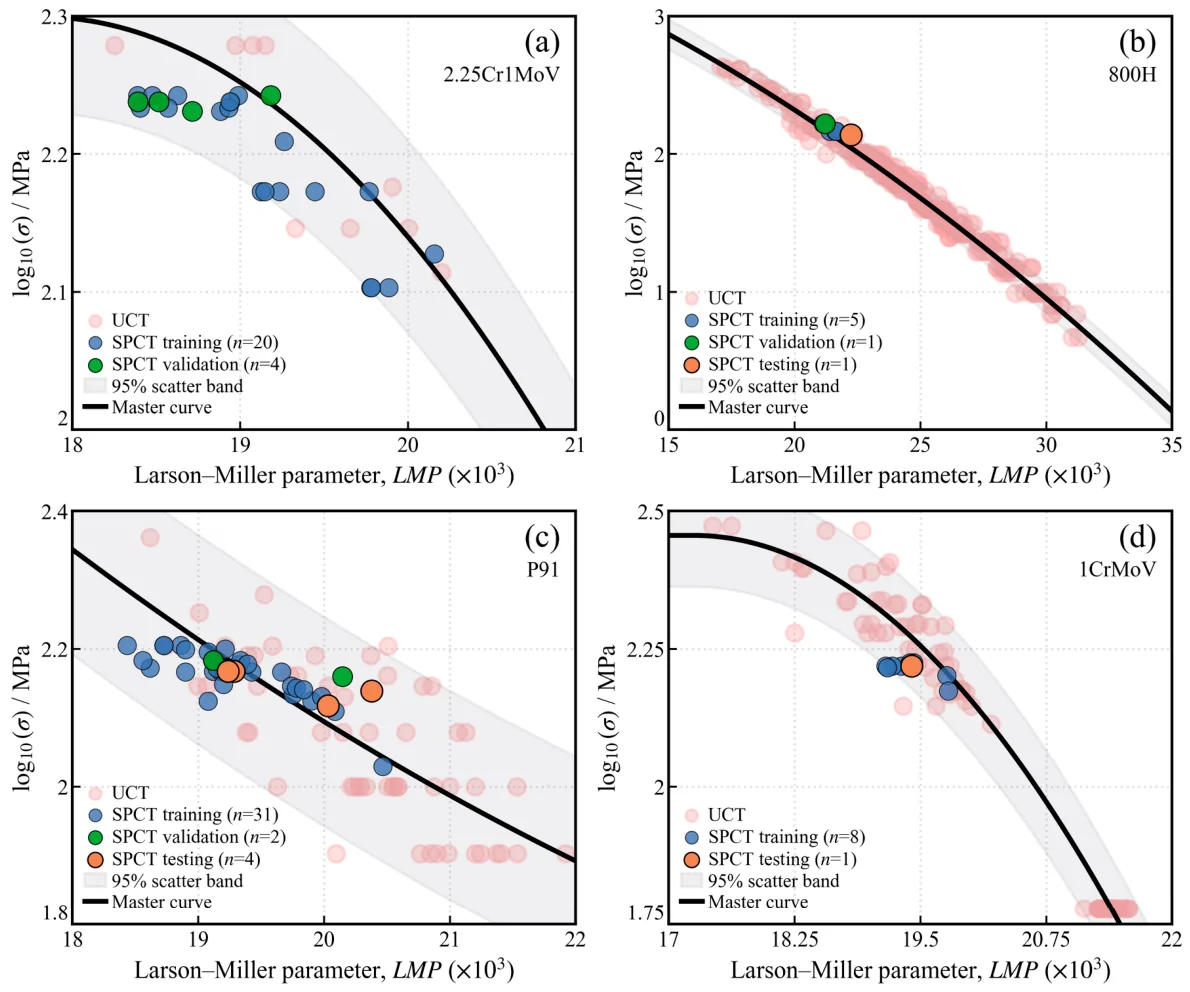
Comparison of creep stress obtained by UCT and SPCT: (**a**) 2.25CrMoV steel, (**b**) Incoloy 800H alloy, (**c**) P91 steel, (**d**) 1CrMoV steel.

Figure 6 presents the evolution of the training and validation losses of the Mapper Model. The shaded regions (±1 standard deviation) are remarkably tight. The mean losses decrease rapidly during the initial training stage and subsequently approach a plateau. After 2500 epochs, the validation loss stabilizes at approximately 2.6. This convergence behavior indicates that the Mapper Network has obtained a favorable parameter solution for mapping the SPCT force to the equivalent uniaxial creep stress. Moreover, the smooth and stable training process validates the proposed warm-start initialization strategy and the optimization framework. Figure 7 compares the equivalent uniaxial stresses converted from SPCT with the Larson–Miller master curves of the corresponding uniaxial creep tests. The horizontal axis is the Larson–Miller parameter with *C* = 20, and the vertical axis is the logarithm of stress. Each panel shows the uniaxial data, the fitted master curve, and the 95% scatter band of that curve. The SPCT points are the mean equivalent stresses from 20 runs of the Mapper Network. Panels (a–d) are 2.25Cr1MoV steel, Incoloy 800H, P91 steel, and 1CrMoV steel. In all four panels, the converted stresses follow the uniaxial master curve, and most points fall within the 95% scatter band.

### 3.3. Factors Influencing Force-to-Stress Ratio

**Figure 8:**
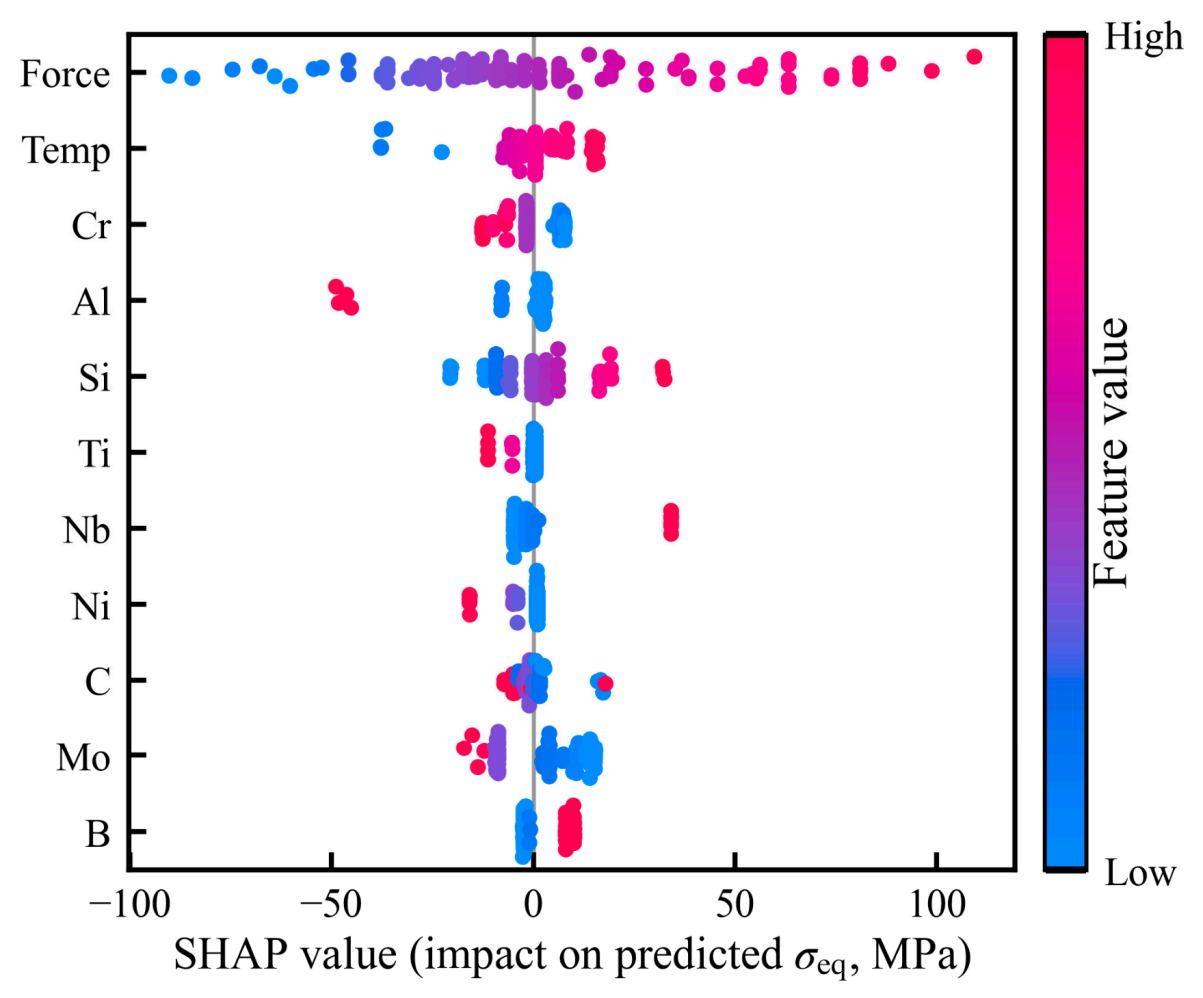
Influences of input features on predicted equivalent uniaxial creep stress.

Figure 8 shows the SHAP results for the Mapper Network, whose output is the equivalent uniaxial creep stress [28]. It can be seen that the SPCT force is the most influential input feature. A high force generates a positive SHAP value, demonstrating that the predicted creep stress increases consistently with the applied SPCT force. This is widely accepted in studies of SPCT [29,30]. Temperature ranks as the second most significant factor, and an increase in testing temperature tends to elevate the creep stress level in SPCT. In addition, the chemical composition of the material also affects the creep stress.

**Figure 9:**
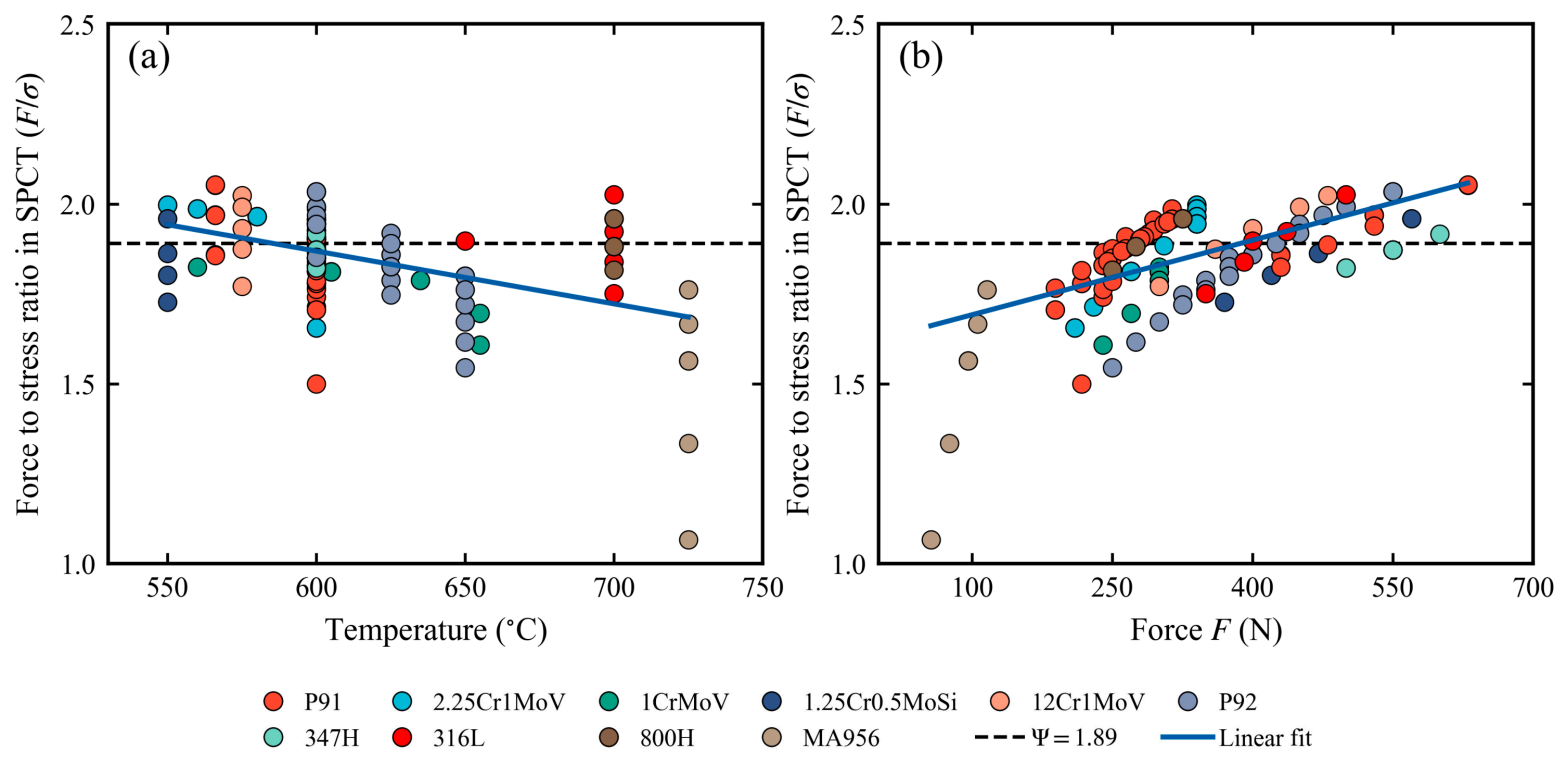
Influences of temperature and SPCT force on force-to-stress ratio: (**a**) temperature and (**b**) SPCT force.

**Figure 10:**
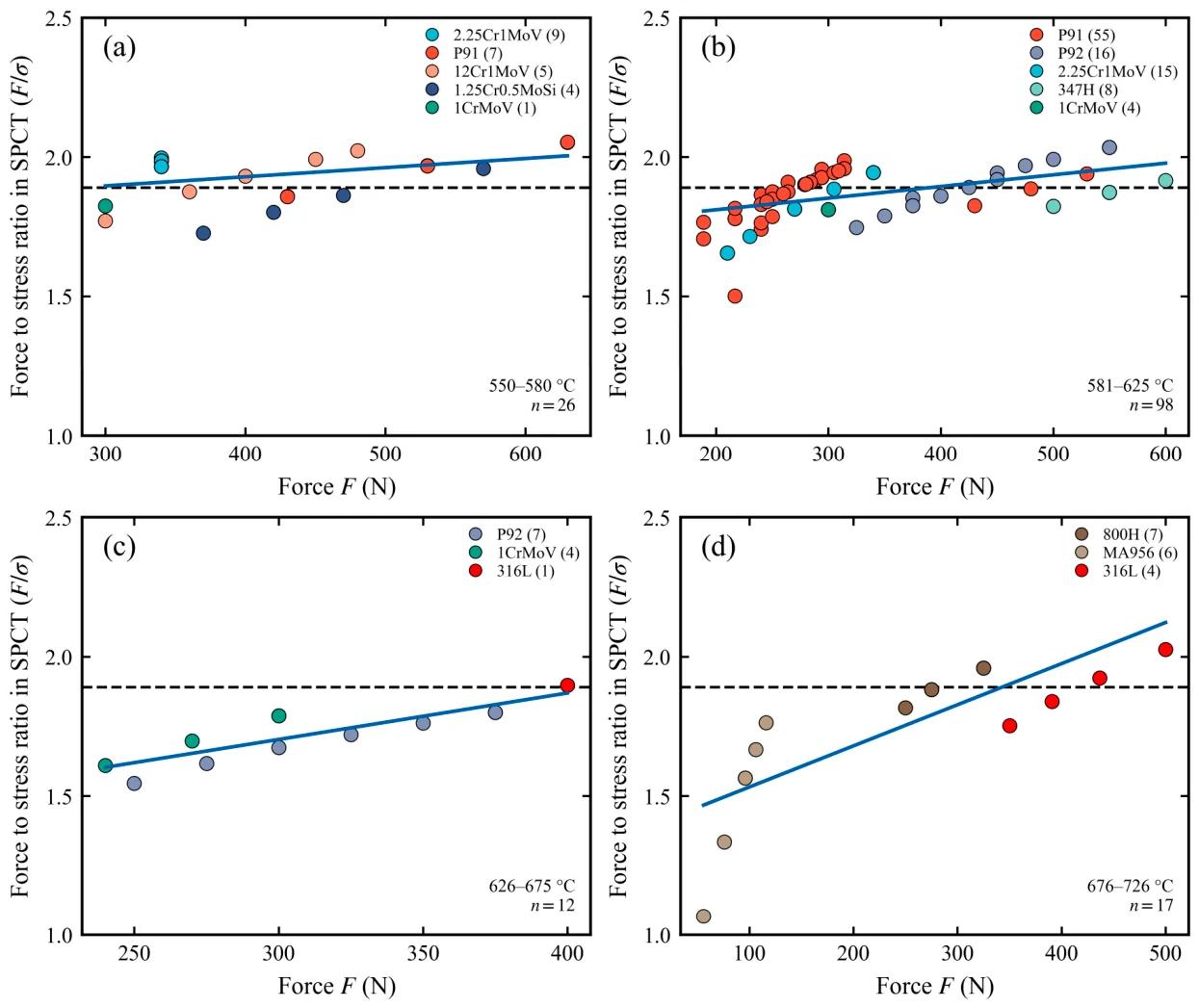
Dependence of force-to-stress ratio on SPCT force at different temperature intervals: (**a**) 550–580 °C, (**b**) 581–625 °C, (**c**) 626–675 °C, and (**d**) 676–725 °C. The solid blue line is the linear fit of the force-to-stress ratio against the SPCT force in each temperature interval. The dashed line indicates the constant ratio Ψ = 1.89 recommended by CWA 15627.

The force-to-stress ratio, Ψ, can be obtained as the equivalent uniaxial creep stress has been predicted by the Mapper Network. Figure 9 presents the dependence of Ψ on the testing temperature and the SPCT force. The black dashed line represents the fixed force-to-stress ratio (Ψ=1.89) recommended by standard CWA-15627. The solid blue line is the linear regression of the predicted Ψ values against testing temperature or force. Figure 9a shows Ψ is not constant and exhibits a pronounced temperature dependence. Particularly at temperatures above 600 °C, for the majority of samples Ψ is lower than 1.89, dropping even below 1.80 under some conditions. Figure 9b shows that Ψ generally increases with increasing SPCT force. At a low force, Ψ is below the empirical value of 1.89, whereas it gradually approaches or slightly exceeds 1.89 at a higher force. Different colors in Figure 9 identify the materials. The fitted lines summarize this mixed set. To further distinguish the effect of SPCT force, the samples were divided into four fixed temperature intervals. The relationship between Ψ and force was examined separately, as shown in Figure 10. It is observed that Ψ increases with increasing force across all four temperature intervals.

The force dependence is relatively weak in the lower-temperature intervals of 550–580 °C and 581–625 °C, with fitted slopes of 0.0003 and 0.0004, respectively. In contrast, the dependence becomes more pronounced at 626–675 °C and 676–725 °C, where the fitted slopes increase to 0.0017 and 0.0015. The results indicate that Ψ is dependent on the SPCT force, and this dependence is temperature-related. Specifically, the influence of force on Ψ becomes more pronounced at high temperature. The differences in the fitted slopes may also reflect which alloys were tested in each interval. These relationships are observed trends from the present datasets; since temperature, force, and material were not varied independently in the experimental matrix, they may not necessarily represent general relationships applicable to other materials and testing conditions.

For a constant applied force, membrane stretching theory suggests that the creep stress of the SPCT specimen is reduced as deflection increases [31,32]. An increase in the applied force leads to a larger initial deflection of the specimen. This raises the creep stress, but the increase in creep stress is less than proportional to the increase in the applied force. Thus, Ψ increases with increasing applied force. The temperature dependence of Ψ may be largely attributed to the fundamentally different stress state between SPCT and UCT. UCT produces an approximately uniform uniaxial stress in the gauge section, whereas SPCT generates a non-uniform and multiaxial stress field. To predict the rupture time of the material under the multiaxial stress state, different expressions of representative rupture stress have been proposed [33,34,35,36]. The representative rupture stress can be understood as the uniaxial stress level on a UCT specimen that is equivalent, in terms of rupture life, to a multiaxial stress state at a given temperature. In other words, it serves as the correlation criterion between uniaxial and multiaxial stress conditions. One of the widely employed expressions is proposed by Hayhurst et al. [33], written as:

(5) $$\sigma_{f}\;=\;\alpha \sigma_{1}\;+\;\left(1\;-\;\alpha \right)\sigma_{e}$$

where $\sigma_{f}$ is the representative rupture stress, which lies between the maximum principal stress $\sigma_{1}$ and the Von Mises stress $\sigma_{e}$. α denotes the multiaxial factor ranging from 0 to 1. In UCT, the maximum principal stress is identical to the Von Mises stress. The representative rupture stress is equal to the maximum principal stress. Nevertheless, the SPCT specimen undergoes a multiaxial stress state. The representative rupture stress is associated not only with the maximum principal stress and equivalent stress, but also with the multiaxial factor. The multiaxial factor is employed to characterize the contribution of different stress components to creep damage under multiaxial stress states. A value of α = 1 indicates that creep damage is predominantly governed by the maximum principal stress, whereas α = 0 signifies that it is entirely controlled by the equivalent stress. Studies have reported that this factor is strongly dependent on the creep failure mechanism, stress level, temperature, and others [37,38,39]. The multiaxial factor increases when the dominant creep failure mode transitions from transgranular cracking to intergranular cracking [37]. Experimental evidence suggests that the creep failure mechanism can shift from transgranular to intergranular by increasing temperature or decreasing stress [40,41]. Since the maximum principal stress in SPCT specimens remains higher than the equivalent stress, the representative rupture stress increases with the multiaxial factor. Therefore, with the SPCT force and UCT stress held constant, an increase in temperature alone can lead to a higher representative rupture stress for SPCT specimens, whereas that for the UCT specimens remains unchanged. According to the definitions of the equivalent uniaxial creep stress derived from SPCT and the representative rupture stress, the two can be considered approximately equivalent. Therefore, an increase in temperature leads to a decrease in Ψ. We emphasize that this explanation is a possible interpretation based on previously reported representative-stress and failure mode arguments [33,34,35,36,37,38,39,40,41], rather than a mechanism directly verified by new measurements in this study.

Figure 8 reveals that the chemical composition of the material affects the output stress of the Mapper Network, implying that Ψ also varies with the material. This can be clarified from two aspects. First, the resistance to deformation varies among different materials. When subjected to identical loading conditions in the SPCT, specimens fabricated from distinct materials manifest disparate deflection behaviors. This leads to varied creep stress states. Second, the multiaxial factor not only depends on the creep failure mechanism, but also a material-related parameter [39,42,43,44]. Thus, the representative rupture stress differs even if the same force is applied on SPCT specimens of different materials. An analytical solution for force-to-creep stress conversion has long been a central challenge in SPCT research. The present results indicate that the force-to-creep stress ratio is significantly influenced by temperature, applied force, and material. Therefore, future studies should not be limited to modifications at the geometrical level, but should pay greater attention to the underlying physical creep mechanisms involved in the SPCT.

## 4. Conclusions

This study proposed a physics-informed framework for converting the small punch creep test (SPCT) force into equivalent uniaxial creep stress for creep-resistant alloys at high temperatures. The main conclusions drawn are as follows:

1. A transfer learning framework was proposed to correlate the SPCT force with equivalent uniaxial creep stress. This framework was established using 825 sets of uniaxial creep test data and 153 sets of SPCT data. Unlike existing methods, this approach considered the effects of testing temperature and materials on the creep mechanisms in SPCT, and realized a nonlinear force-to-stress correlation.
2. The Larson–Miller comparison uses P91 steel, 2.25Cr1MoV steel, 1CrMoV steel, and Incoloy 800H. The equivalent stresses obtained from SPCT follow the uniaxial master curves, and most points lie within the 95% scatter of those curves. Each converted stress is the mean of 20 runs.
3. In the present dataset, the force-to-stress ratio decreases with increasing temperature and increases with applied force within the tested range; these observed trends may not necessarily apply to other materials and testing conditions. In the SHAP results, force accounts for the largest share of the predicted stress, followed by temperature and chemical composition. The SPCT specimens are 0.5 mm thick. Under the membrane relation used for this specimen geometry, the stress corresponding to a given force varies approximately as $1/t^{2}$, and the ratios reported here belong to this thickness.

## Figures and Tables

**Table 1:** Network architectures and training hyperparameters of the Teacher Model and Mapper Network.

Item	Teacher Model	Mapper Network
Input dimension	11	11
Hidden layers	64–64–32	32–32
Activation	ReLU	ReLU
Output activation	Linear	Softplus
Optimizer	Adam	Adam
Learning rate	0.001	0.005
Batch size	Full batch (825)	Full batch (153)
Epochs	1500	2500
Random seeds	20	20

**Table 2:** Statistical summary of the uniaxial creep dataset (source domain).

Feature	Unit	Min	Max	Mean	SD
Temperature	°C	450	1150	700.6	152.1
Stress	MPa	4.7	900	171.7	139.7
Rupture time	h	10	132,550	5484.2	13,689.4
Ni	wt.%	0	77	23.16	24.76
Al	wt.%	0	5.97	0.768	1.388
Cr	wt.%	0.36	25	12.38	8.65
Mo	wt.%	0	5.7	0.787	0.772
Ti	wt.%	0	3.9	0.817	1.372
Nb	wt.%	0	5.1	0.209	0.492
Si	wt.%	0	1	0.313	0.209
C	wt.%	0	0.22	0.096	0.044
B	wt.%	0	0.02	0.002	0.004

**Table 3:** Statistical summary of the small punch creep dataset (target domain).

Feature	Unit	Min	Max	Mean	SD
Temperature	°C	550	725	613.5	39.74
Force	N	56	630	330.1	115.5
Rupture Time	h	10.81	4150	449.5	914.2
C	wt.%	0.011	0.300	0.108	0.045
Si	wt.%	0.130	0.680	0.280	0.120
Cr	wt.%	0.000	19.970	8.800	5.670
Mo	wt.%	0.000	2.640	0.770	0.520
Ni	wt.%	0.000	30.880	2.533	6.932
Al	wt.%	0.000	4.500	0.205	0.890
Ti	wt.%	0.000	0.440	0.080	0.140
Nb	wt.%	0.000	0.510	0.080	0.120
B	wt.%	0.000	0.003	0.001	0.001

## Data Availability

The data presented in this study are available on request from the corresponding author. The data are not publicly available due to privacy restrictions.
